# Liquid Crystal Phase Behaviour of Attractive Disc-Like Particles

**DOI:** 10.3390/ijms140816414

**Published:** 2013-08-08

**Authors:** Liang Wu, George Jackson, Erich A. Müller

**Affiliations:** Department of Chemical Engineering, Imperial College London, South Kensington Campus, London SW7 2AZ, UK; E-Mails: l.wu09@imperial.ac.uk (L.W.); g.jackson@imperial.ac.uk (G.J.)

**Keywords:** equation of state, discotics, attractive cylindrical disc, nematic-nematic equilibria, anisotropic square well, phase diagrams

## Abstract

We employ a generalized van der Waals-Onsager perturbation theory to construct a free energy functional capable of describing the thermodynamic properties and orientational order of the isotropic and nematic phases of attractive disc particles. The model mesogen is a hard (purely repulsive) cylindrical disc particle decorated with an anisotropic square-well attractive potential placed at the centre of mass. Even for isotropic attractive interactions, the resulting overall inter-particle potential is anisotropic, due to the orientation-dependent excluded volume of the underlying hard core. An algebraic equation of state for attractive disc particles is developed by adopting the Onsager trial function to characterize the orientational order in the nematic phase. The theory is then used to represent the fluid-phase behaviour (vapour-liquid, isotropic-nematic, and nematic-nematic) of the oblate attractive particles for varying values of the molecular aspect ratio and parameters of the attractive potential. When compared to the phase diagram of their athermal analogues, it is seen that the addition of an attractive interaction facilitates the formation of orientationally-ordered phases. Most interestingly, for certain aspect ratios, a coexistence between two anisotropic nematic phases is exhibited by the attractive disc-like fluids.

## 1. Introduction

Liquid crystals [1–3] are intermediate (meso) phases with characteristics that lie between the fully positionally- and orientationally-ordered crystal and the disordered liquid states. Since their discovery by Reinitzer in 1883 [4], liquid crystals (LCs) have attracted a longstanding research interest, because of their unique thermodynamic, structural, optical and electronic properties. An essential requirement for the stabilization of an LC phase is that molecules be highly anisometric in shape. A wide variety of oblate disc-shaped LC particles with length scales ranging from tens of Ångström (molecular dimensions) to hundreds of nanometers (macromolecular/colloidal dimensions) have been observed to exhibit rich phase behaviour, including isotropic, nematic and columnar phases.

Discotic LCs tend to comprise poly-aromatic cores of oblate geometry, e.g., hexa-alkanoyloxy benzenes, or metal organic complexes of phenyl pyridines, porphyrazines, phthalocyanines, triphenylenes, *etc*. [2,3,5–7]. These relatively low molecular weight compounds are commonly referred to as thermotropic liquid crystals, owing to the leading role temperature plays in the phase transformations between the various states. Colloidal particles of discotic geometry have also been extensively studied in recent years, including nickel hydroxide theophrastite sheets, aluminum hydroxide gibbsite platelets and nontronite or laponite mineral clays [8–16]. The stability of the colloidal anisotropic phases are commonly governed by the solute concentration, a characteristic that leads to the classification of such systems as lyotropic LCs. An interesting intermediate class of naturally occurring discotic particles includes asphaltenes, polyaromatic compounds of intermediate molecular weight (500 to 10,000 g/mol) found in heavy crude oils, which aggregate and precipitate from solution over particular ranges of composition, temperature and pressure [17]. As a result of their oblate rigid cores, these asphaltenic systems exhibit anisotropic LC phases with both thermotropic and lyotropic characteristics [18–22].

In computer simulation studies [23–36] and theoretical treatments [37–46], simple disc-like particles are often taken as prototypical models of the oblate LC molecules; the discs are usually characterized by their aspect ratio, *D/L*, where *L* is the thickness of the disc and *D* is its diameter. Hard disc-like particles have proven to be appropriate models for lyotropic colloidal LCs.

The theoretical treatment of hard non-spherical fluids dates back to the 1940s [8]. The Onsager view that repulsive (excluded volume) interactions are of key importance in the formation of orientationally ordered phases is now widely accepted. In his pioneering work on nematic LCs [8], Onsager proposed the now well-accepted free energy functional for nematic states and demonstrated that the isotropic-nematic phase transition can be driven by entropy alone. Entropy-driven phase transitions have now been studied extensively by computer simulation of hard-body anisometric particles, where both nematic (discotic) and columnar ordering has been identified in hard-disc models upon increasing the density of the system [23–25,27,28,33–36,47].

For a proper description of the temperature dependence of the thermodynamic properties of a system, both repulsive and attractive inter-molecular interactions have to be considered on an equal footing. This is in line with a van der Waals description of fluids, where the hard core is treated as the reference in determining the fluid structure [48,49], and a mean-field attraction is included to account for the cohesive interactions and fluid-phase equilibria. A simple hard-core model which incorporates the attractive interactions therefore offers promise in representing the phase behaviour of thermotropic LCs. A number of studies have extended the early work of Kimura [50], who combined Onsager’s hard-rod model with anisotropic dispersion forces (e.g., see [51–60]). Some further progress has been made in understanding the effect of generalized attractive potentials [60–64] and dipolar [65–70] or chiral [71–73] interactions on the stability of the various LC phases.

As has already been mentioned, purely repulsive discotic particles have been shown to exhibit columnar phases in the higher density region. This was first demonstrated by Veerman and Frenkel [25,74] with simulations of the hard cut-sphere system; columnar order was then subsequently found in systems of oblate hard spherocylinders [32]. In a previous paper [46], we coupled a generic equation of state for the nematic phase of hard disc-like particles with a cell theory [39,44] to describe the boundaries of the isotropic, nematic, and columnar phases of the hard cut spheres. The transition from a nematic to a columnar state is observed at packing fractions of about 40%, and the coexistence densities are found to be relatively insensitive to the aspect ratio of the discs (apart, perhaps, in the limit of infinitely thin particles). This means that for hard discs in dense states above a ~40% packing fraction, the columnar phase (and other positionally ordered phases such as the crystalline solid) will be stable relative to the nematic state. In the case of discs with attractive interactions, one may also expect an additional stabilization of the columnar phase, depending on the nature of the attractions; e.g., oblate Gay-Berne particles exhibit a deep energetic minimum when the particles are close together in a face-to-face parallel relative orientation, leading to an enhanced stability of the columnar structure [75] compared to the lack of a columnar phase for purely repulsive oblate ellipsoids [76]. For our model hard discs with square-well attractions, however, the nature of the enveloping square well is such that multiple energetic contacts are possible when the particles are not directly over one another, so that the energetic gain from a columnar geometry is not as evident (cf. the work of del Rio *et al.* [63] for disc particles with large ranges of the attractive well). As a consequence, we would not expect discs with enveloping attractive wells to exhibit columnar phases for states of low to moderate density, 0 *< η <* 0.35, though caution has to be exercised in assessing the stability of the nematic phase for the higher density states. It would be interesting to simulate such systems to confirm this, but such an analysis is beyond the scope of the current work.

**Figure 1:**
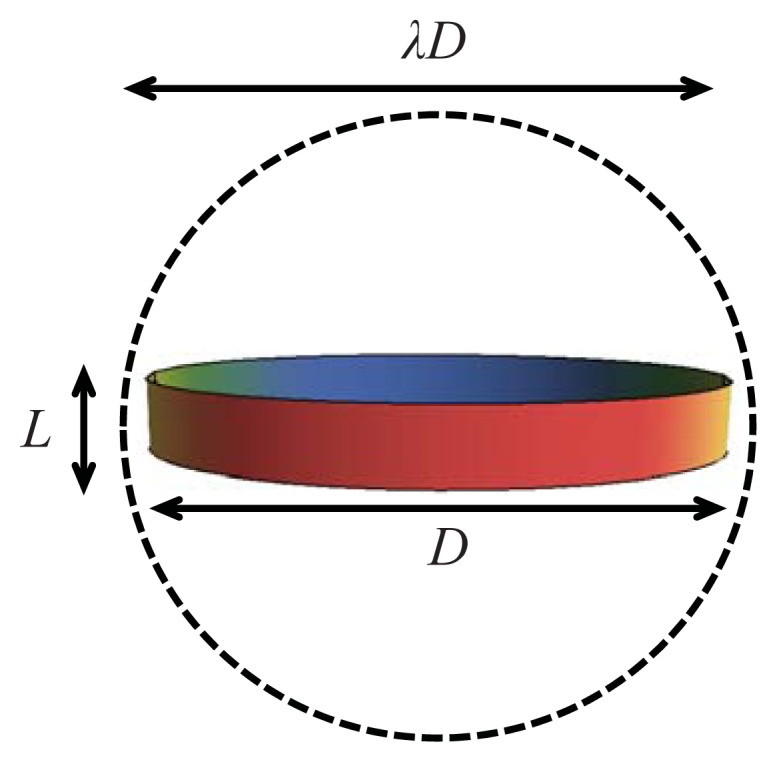
The attractive cylindrical disc (ACD) model. The model is characterized by the thickness, *L*, and diameter, *D*, of the hard core. A spherical attractive interaction of range *λD* is represented as the dashed sphere.

Of particular importance here is the closed-form algebraic equation of state (EOS) developed for attractive hard spherocylinders [77,78], where the attractive potential is expanded in spherical harmonics representing different contributions to the anisotropic attractions [79]. We develop a model for attractive oblate (cylindrical-disc) mesogens by adding an anisotropic square-well (SW) potential, which mediates the intermolecular attractions, to a hard-cylindrical disc. The model of attractive discs is depicted in Figure 1, where the parameter *λ* is introduced to quantify the attractive range. A perturbation approach is applied to develop a free energy functional for the attractive disc system. By using the Onsager trial function to describe the degree of orientational order in the nematic phase, the free energy and equation of state for the nematic state can be expressed in closed algebraic form, which also allows a straightforward superimposition of contributions from intermolecular interactions such as chiral, dipolar and associating interactions, or the extension to other types of dispersive intermolecular potentials (e.g., Lennard-Jones, Mie, Yukawa).

## 2. Theory

### 2.1. Generalized van der Waals-Onsager Free Energy Functional

In this section, we describe the perturbation theory [49,80] employed to construct the free energy for the nematic and isotropic phases of attractive non-spherical particles. A system of *N* particles in a volume *V* at a temperature *T* is considered, where the total Helmholtz free energy, *A*, is divided into two contributions, a reference term and a perturbation term. The former is assumed to be known precisely, and the latter represents the contribution from the attractive potential perturbation:

(1) $$\frac{A}{NkT}=\frac{A^{\text{ref}}}{NkT}+\frac{A^{\text{att}}}{NkT}$$

Here, *k* is the Boltzmann constant, and the reference contribution, *A*^ref^, is taken from an expression for the hard-core particles. Following the Parsons-Lee approach (PL) [81–83], the reference repulsive term can be expressed in compact form as:

(2) $$\frac{A^{\text{ref}}}{NkT}=\frac{A_{\text{iso}}^{\text{id}}}{NkT}+\int f(\vec{\omega })\;\text{ln }\{4\pi f(\vec{\omega })\}\text{d}\vec{\omega }+G(\eta )\iint V_{\text{exc}}({\vec{\omega }}_{1},{\vec{\omega }}_{2})f({\vec{\omega }}_{1})f({\vec{\omega }}_{2})\text{d}{\vec{\omega }}_{1}\text{d}{\vec{\omega }}_{2}$$

where $A_{\text{iso}}^{\text{id}}$ is the ideal contribution to the free energy of the isotropic state (incorporating the translational and rotational kinetic terms), *ω⃗* is the orientation vector of a particle, and *f*(*ω⃗*) is the single-particle orientational distribution function. In the Parsons-Lee approach, the contributions from the high-order virial terms are incorporated in an effective manner by scaling the corresponding hard-sphere equation of state [84,85]. The density-dependent function, *G*(*η*), represents the residual free energy of the equivalent hard-sphere system:

(3) $$G(\eta )=\frac{4\eta -3\eta^{2}}{8V_{\text{m}}{\left(1-\eta \right)}^{2}}$$

where *η* = *ρV*_m_ is the packing fraction, *ρ* is the single-particle density and *V*_m_ is the molecular volume. Alternatively, a improved generic equation of state for hard disc-like particles developed in [46] can be chosen to describe the reference system.

In the high-temperature limit, the attractive free energy can be approximated at first order [49,77,80] by:

(4) $$A^{\text{att}}={\langle U^{\text{att}}\rangle }_{\text{ref}}$$

where *<* ···*>*_ref_ represents an ensemble average over all configurations of the reference system. In the canonical ensemble, the mean-attractive energy is formally written as:

(5) $$\begin{array}{l} A^{\text{att}}\approx {\langle U^{\text{att}}\rangle }_{\text{ref}}\\ =\frac{1}{Z_{NVT}}\iint \text{d}{\vec{r}}^{N}\text{d}{\vec{\omega }}^{N}U^{\text{att}}({\vec{r}}^{N},{\vec{\omega }}^{N})\;\text{exp }\left(-\frac{U^{\text{ref}}({\vec{r}}^{N},{\vec{\omega }}^{N})}{kT}\right) \end{array}$$

with the configurational integral defined as:

(6) $$Z_{NVT}=\iint \text{d}{\vec{r}}^{N}\text{d}{\vec{\omega }}^{N}\;\text{exp }\left(-\frac{U^{\text{ref}}({\vec{r}}^{N},{\vec{\omega }}^{N})}{kT}\right)$$

which is a function of the positions, *⇉^N^*, and orientations, *ω⃗^N^*, of all *N* particles. Further simplification is achieved by assuming pairwise additive interactions, such that $U^{\text{att}}({\vec{r}}^{N},{\vec{\omega }}^{N})=\sum_{i=1}^{N}\sum_{j>i}^{N}u_{ij}^{\text{att}}\;({\vec{r}}_{ij},{\vec{\omega }}_{i},{\vec{\omega }}_{j})$, where the vector *⇉_ij_* = *⇉_i_*−*⇉_j_*, and introducing the pair distribution function of reference system, *g*^ref^ (*⇉*_12_, *ω⃗*_1_, *ω⃗*_2_), defined as:

(7) $$\begin{array}{l} g^{\text{ref}}({\vec{r}}_{12},{\vec{\omega }}_{1},{\vec{\omega }}_{2})=\frac{\rho_{12}^{\text{ref}}({\vec{r}}_{12},{\vec{\omega }}_{1},{\vec{\omega }}_{2})}{\rho_{1}^{\text{ref}}({\vec{r}}_{1},{\vec{\omega }}_{1})\rho_{1}^{\text{ref}}({\vec{r}}_{2},{\vec{\omega }}_{2})}\\ =\frac{N(N-1)}{\rho_{1}^{\text{ref}}({\vec{r}}_{1},{\vec{\omega }}_{1})\rho_{1}^{\text{ref}}({\vec{r}}_{2},{\vec{\omega }}_{2})}\times \frac{1}{Z_{\text{NVT}}}\int \text{d}{\vec{r}}^{N-2}\text{d}{\vec{\omega }}^{N-2}\;\text{exp }\left[-\frac{U^{\text{ref}}({\vec{r}}^{N},{\vec{\omega }}^{N})}{kT}\right] \end{array}$$

The attractive term can thus be re-written as a sum of pair contributions. When considering a nematic phase where the distribution of particle positions is uniform, the single-particle density of the system can be factorised as *ρ*^ref^(*⇉_i_, ω⃗_i_*) = *ρf*(*ω⃗_i_*), where the single-particle orientation distribution function, *f*(*ω⃗*), has been introduced. On inserting Equation (7) into Equation (5) and using the nematic form of the single-particle density, one can write:

(8) $$\begin{array}{l} A^{\text{att}}=\frac{1}{2}\int \int \int \int u_{12}^{\text{att}}\;({\vec{r}}_{12},{\vec{\omega }}_{1},{\vec{\omega }}_{2})\;g^{\text{ref}}({\vec{r}}_{12},{\vec{\omega }}_{1},{\vec{\omega }}_{2})\times \rho^{\text{ref}}({\vec{r}}_{1},{\vec{\omega }}_{1})\;\rho^{\text{ref}}\;({\vec{r}}_{2},{\vec{\omega }}_{2})\;\text{d}{\vec{r}}_{1}\text{d}{\vec{\omega }}_{1}\text{d}{\vec{r}}_{2}\text{d}{\vec{\omega }}_{2}\\ =\frac{\rho^{2}V}{2}∭u_{12}^{\text{att}}\;({\vec{r}}_{12},{\vec{\omega }}_{1},{\vec{\omega }}_{2})\times f({\vec{\omega }}_{1})\;f({\vec{\omega }}_{2})g^{\text{ref}}\;({\vec{r}}_{12},{\vec{\omega }}_{1},{\vec{\omega }}_{2})\;\text{d}{\vec{r}}_{12}\text{d}{\vec{\omega }}_{1}\text{d}{\vec{\omega }}_{2} \end{array}$$

Since the particles of interest are non-spherical, both the attractive interaction and the excluded volume are complicated functions of the relative positions and orientations of particles [79,86]. It is thus useful to expand the pair potential as a series in spherical harmonics. Here, we consider an anisotropic square-well (ASW) potential of the form [77,79]:

(9) $$u^{\text{att}}({\vec{r}}_{12},{\vec{\omega }}_{1},{\vec{\omega }}_{2})=-s(r_{12})\;[{ɛ}_{0}+{ɛ}_{2}P_{2}(\text{cos }\gamma )]$$

(10) $$s(r_{12})=\{\begin{array}{ll} 1 & \sigma ({\hat{\vec{r}}}_{12},{\vec{\omega }}_{1},{\vec{\omega }}_{2})\geq r_{12}<\lambda D\\ 0 & r_{12}\geq \lambda D \end{array}$$

where *λ* is the range parameter, *D* is the reference diameter of the disc, and *P*_2_(cos *γ*) is the second Legendre polynomial for the relative orientation, *γ* = arccos(*ω⃗*_1_ · *ω⃗*_2_), between the principle axes of the two discs. In order to make the approach tractable, we use the low-density limit to approximate the pair distribution function of the reference system:

(11) $$\underset{\rho \to 0}{\text{lim}}\;g^{\text{ref}}({\vec{r}}_{12},{\vec{\omega }}_{1},{\vec{\omega }}_{2})=\text{exp }\left(-\frac{u^{\text{ref}}({\vec{r}}_{12},{\vec{\omega }}_{1},{\vec{\omega }}_{2})}{kT}\right)$$

where *u*^ref^ (*⇉*_12_, *ω⃗*_1_, *ω⃗*_2_) is the pair interaction of the reference system. Since the reference system corresponds to the hard-core particles, *u*^ref^ (*⇉*_12_, *ω⃗*_1_, *ω⃗*_2_) is a purely repulsive interaction, which is infinity when hard cores overlap, and zero otherwise. Introducing the ASW attractive potential in Equation (8) with the approximation of Equation (11) and integrating out the centre-to-centre distance between particle 1 and 2, one can express the free energy perturbation as [77,78]:

(12) $$\begin{array}{l} \frac{A^{\text{att}}}{NkT}=-\frac{\rho {ɛ}_{0}}{2kT}\;\left[\frac{4\pi }{3}\lambda^{3}\;D^{3}-{\langle V_{\text{exc}}(\gamma )\rangle }_{{\vec{\omega }}_{1},{\vec{\omega }}_{2}}\right]\\ -\frac{\rho {ɛ}_{2}}{2kT}\;\left[\frac{4\pi }{3}\lambda^{3}\;D^{3}{\langle P_{2}(\text{cos}\;\gamma )\rangle }_{{\vec{\omega }}_{1},{\vec{\omega }}_{2}}-{\langle V_{\text{exc}}(\gamma )P_{2}(\text{cos }\gamma )\rangle }_{{\vec{\omega }}_{1},{\vec{\omega }}_{2}}\right] \end{array}$$

where the double orientational average of a quantity *J*(*ω⃗*_1_, *ω⃗*_2_) is defined as:

(13) $${\langle J({\vec{\omega }}_{1},{\vec{\omega }}_{2})\rangle }_{{\vec{\omega }}_{1},{\vec{\omega }}_{2}}=\iint J({\vec{\omega }}_{1},{\vec{\omega }}_{2})f({\vec{\omega }}_{1})f({\vec{\omega }}_{2})\text{d}{\vec{\omega }}_{1}\text{d}{\vec{\omega }}_{2}$$

Combining the separate contributions, the free energy of the full system is obtained in terms of angle averages of the configurational contributions:

(14) $$\begin{array}{l} \frac{A[f(\vec{\omega })]}{NkT}=\frac{A_{\text{iso}}^{\text{id}}}{NkT}+\int f(\vec{\omega })\;\text{ln }[\Omega f(\vec{\omega })]\;\text{d}\vec{\omega }+G(\eta )\;{\langle V_{\text{exc}}(\gamma )\rangle }_{{\vec{\omega }}_{1},{\vec{\omega }}_{2}}\\ -\frac{\rho {ɛ}_{0}}{2kT}\;\left[\frac{4\pi }{3}\lambda^{3}\;D^{3}-{\langle V_{\text{exc}}(\gamma )\rangle }_{{\vec{\omega }}_{1},{\vec{\omega }}_{2}}\right]\\ -\frac{\rho {ɛ}_{2}}{2kT}\;\left[\frac{4\pi }{3}\lambda^{3}\;D^{3}{\langle P_{2}(\text{cos}\;\gamma )\rangle }_{{\vec{\omega }}_{1},{\vec{\omega }}_{2}}-{\langle V_{\text{exc}}(\gamma )P_{2}(\text{cos}\;\gamma )\rangle }_{{\vec{\omega }}_{1},{\vec{\omega }}_{2}}\right] \end{array}$$

In this expression for the free energy, one can recognize the coupling between the repulsive and attractive contributions of the pair potential. The excluded volume is seen in both the terms corresponding to the isotropic and the anisotropic attractive contributions; the last term in 〈*V*_exc_(*γ*)*P*_2_(cos *γ*)〉*_ω⃗_*__1_,_*_ω⃗_*__2__ also constitutes a direct coupling of the two contributions. The single-particle orientational distribution function in the isotropic state is constant, *f*_iso_(*ω⃗*) = 1/4*π*, which simplifies the orientational integrals of Equation (14). In the anisotropic phases, the equilibrium orientational distribution of the molecules can be found by minimizing the total free energy functional, *A* [*f*(*ω⃗*)].

### 2.2. Equation of State for Hard-Cylindrical Disc Particles with an Anisotropic Square-Well Potential

We start by examining the angle averages of the purely repulsive contributions, *i.e*., the excluded volume term. The excluded volume, *V*_exc_(*γ*), of hard cylinders can be expressed conveniently as a power series in sin *γ*:

(15) $$V_{\text{exc}}(\gamma )=\sum_{i=0}^{\infty }C_{i}\;{\text{sin}}^{i}\gamma$$

where the coefficient, *C_i_*, depends on the specific geometry of molecules. It had been shown that an approximate expression, which is obtained by truncation of the series at the fourth-order term in sin^4^*γ*, is sufficient to faithfully reproduce exact numerical results [46]. The excluded volume of two hard cylinders can be accurately represented as [46,87]:

(16) $$\frac{V_{\text{exc}}(\gamma )}{V_{\text{m}}}=C_{0}^{*}+C_{1}^{*}\;\text{sin }\gamma +C_{2}^{*}\;{\text{sin}}^{2}\;\gamma +C_{4}^{*}\;{\text{sin}}^{4}\;\gamma$$

where the coefficients are given by:

(17) $$\begin{array}{ll} C_{0}^{*}=8, & C_{1}^{*}=2\frac{D}{L}+\frac{8}{\pi }\frac{L}{D}\\ C_{2}^{*}=\frac{3\pi }{4}+\frac{8}{\pi }-7, & C_{4}^{*}=(4-3\pi )/16 \end{array}$$

and the molecular volume of a cylindrical disc is *V*_m_ = *πLD*^2^/4. Using the notation of Equation (13), the double orientational average of the excluded volume can be written as:

(18) $${\langle \frac{V_{\text{exc}}(\gamma )}{V_{\text{m}}}\rangle }_{{\vec{\omega }}_{1},{\vec{\omega }}_{2}}=C_{0}^{*}+C_{1}^{*}\;{\langle \text{sin }\gamma \rangle }_{{\vec{\omega }}_{1},{\vec{\omega }}_{2}}C_{2}^{*}\;{\langle {\text{sin}}^{2}\;\gamma \rangle }_{{\vec{\omega }}_{1},{\vec{\omega }}_{2}}+C_{4}^{*}\;{\langle {\text{sin}}^{4}\;\gamma \rangle }_{{\vec{\omega }}_{1},{\vec{\omega }}_{2}}$$

The equilibrium state corresponds to the minimum of the total free energy, *A* [*f*(*ω⃗*)], with respect to the orientational distribution function under the additional constraint that the orientational distribution function is normalised:

(19) $$\frac{\delta }{\delta \;f(\vec{\omega })}(A[f(\vec{\omega })]-\lambda_{L}\int f(\vec{\omega })\text{d}\vec{\omega })=0$$

where *λ*_L_ is a Lagrange undetermined multiplier, which ensures that ∫ *f*(*ω⃗*)d*ω⃗* = 1. The minimum condition leads to a self-consistent integral equation for *f*(*ω⃗*), which can be solved by various numerical methods, including the expansion of the orientational distribution function as a spherical harmonic series [88,89] or use of Monte Carlo annealing techniques [90]. These approaches do not, however, provide an analytical solution for the equilibrium orientational distribution function. In his seminal publication [8], Onsager proposed a functional form for *f*(*ω⃗*) in terms of a parameter *α*, which characterizes the degree of orientational order. This so-called Onsager trial function (OTF) *f*_OTF_(*ω⃗*) is written in hyperbolic form:

(20) $$f_{\text{OTF}}(\vec{\omega })=\frac{\alpha \;\text{cosh}(\alpha \;\text{cos }\theta )}{4\pi \;\text{sinh}(\alpha )}$$

and is seen to depend on the parameter *α*, and the polar angle *θ* = arccos (*ω⃗* · *ω⃗*_0_), where *ω⃗*_0_ is the director of the nematic phase. One should note that when *α* → 0, *f*_OTF_(*ω⃗*) naturally reduces to 1/4*π*, corresponding to the expected distribution in the isotropic phase. Large values of *α* (~10) correspond to a nematic phase. On integrating the OTF over all possible orientations, one can show that it is correctly normalised; thus, it is not necessary to include the term in the multiplier, *λ*_L_, in Equation (19) when incorporating the OTF. The details of calculations of the orientational averages using the OTF have been discussed in detail in earlier publications [78,91]. The introduction of the OTF to describe the degree of orientational order of the nematic phase allows the orientational averages to be rendered in algebraic form, which is more tractable in practice [77,78,91]:

(21) $${\langle \text{sin }\gamma \rangle }_{{\vec{\omega }}_{1},{\vec{\omega }}_{2}}\approx \sqrt{\pi }\left(\frac{1}{\alpha^{1/2}}-\frac{15}{16\alpha^{3/2}}\right)+O(1/\alpha^{5/2})$$

(22) $${\langle {\text{sin}}^{2}\;\gamma \rangle }_{{\vec{\omega }}_{1},{\vec{\omega }}_{2}}\approx \frac{4}{\alpha }+O(1/\alpha^{2})$$

(23) $${\langle {\text{sin}}^{3}\;\gamma \rangle }_{{\vec{\omega }}_{1},{\vec{\omega }}_{2}}\approx \sqrt{\pi }\frac{6}{\alpha^{3/2}}+O(1/\alpha^{5/2})$$

(24) $${\langle {\text{sin}}^{4}\;\gamma \rangle }_{{\vec{\omega }}_{1},{\vec{\omega }}_{2}}\approx 0+O(1/\alpha^{2})$$

Using Equations (21)–(24) in Equation (18) for the excluded volume and *P*_2_(sin *γ*) = 1 − 3 sin_2_*γ/*2 for the anisotropic attractive term, the contributions to configurational free energy can be expressed as:

(25) $${\langle P_{2}(\text{sin }\gamma )\rangle }_{{\vec{\omega }}_{1},{\vec{\omega }}_{2}}\approx 1-\frac{6}{\alpha }$$

(26) $${\langle \frac{V_{\text{exc}}(\gamma )}{V_{\text{m}}}\rangle }_{{\vec{\omega }}_{1},{\vec{\omega }}_{2}}\approx C_{0}^{*}+C_{1}^{*}\sqrt{\pi }\left(\frac{1}{\alpha^{1/2}}-\frac{15}{16\alpha^{3/2}}\right)+C_{2}^{*}\frac{4}{\alpha }$$

(27) $${\langle \frac{V_{\text{exc}}(\gamma )}{V_{\text{m}}}P_{2}(\text{sin }\gamma )\rangle }_{{\vec{\omega }}_{1},{\vec{\omega }}_{2}}\approx C_{0}^{*}+C_{1}^{*}\sqrt{\pi }\left(\frac{1}{\alpha^{1/2}}-\frac{15}{16\alpha^{3/2}}\right)+\left(C_{2}^{*}-\frac{3}{2}C_{0}^{*}\right)\frac{4}{\alpha }-\frac{9}{\alpha^{3/2}}C_{1}^{*}\sqrt{\pi }$$

Collecting all of the terms, one obtains the Helmholtz free energy for the system of attractive cylinder disc (ACD) in terms of the orientational parameter *α*:

(28) $$\begin{array}{l} \frac{A^{\text{nem}}(\alpha )}{NkT}=\frac{A_{\text{iso}}^{\text{id}}}{NkT}+\text{ln }\alpha -1+G(\eta )\;\left[C_{0}^{*}+C_{1}^{*}\sqrt{\pi }\;\left(\frac{1}{\alpha^{1/2}}-\frac{15}{16\alpha^{3/2}}\right)+C_{2}^{*}\frac{4}{\alpha }\right]\\ -\frac{\rho V_{\text{m}}}{2}\frac{{ɛ}_{0}}{kT}\;\{\left(\frac{4\pi }{3}\frac{D^{3}}{V_{\text{m}}}-C_{0}^{*}\right)\;\left(1+\frac{{ɛ}_{2}}{{ɛ}_{0}}\right)-C_{1}^{*}\sqrt{\pi }\;\left(1+\frac{{ɛ}_{2}}{{ɛ}_{0}}\right)\;\alpha^{-1/2}\\ -\left[4C_{2}^{*}\;\left(1+\frac{{ɛ}_{2}}{{ɛ}_{0}}\right)+6\frac{{ɛ}_{2}}{{ɛ}_{0}}\;\left(\frac{4\pi }{3}\frac{D^{3}}{V_{\text{m}}}-C_{0}^{*}\right)\right]\;\alpha^{-1}+\frac{15}{16}C_{1}^{*}\sqrt{\pi }\;\left(1+\frac{53}{5}\frac{{ɛ}_{2}}{{ɛ}_{0}}\right)\;\alpha^{-3/2}\} \end{array}$$

Using the OTF to represent the orientational order of the nematic phase, the free energy is expressed in an explicit algebraic form. As a result, the functional variation of *A* [*f*(*ω⃗*)] with respect to *f*(*ω⃗*) can be simplified to a derivative of the free energy (Equation (28)) with respect to the orientational parameter *α*. Taking the derivative of the free energy with respect to *α* results in a cubic expression for $x=\sqrt{\alpha }$:

(29) $$\frac{\partial }{\partial \alpha }\frac{A(\alpha )}{NkT}=\frac{1}{x^{5}}\;(x^{3}+a_{2}x^{2}+a_{1}x+a_{0})=0$$

where the coefficients are given by:

(30) $$a_{0}=\frac{45}{32}C_{1}^{*}\sqrt{\pi }\;\left[G(\eta )+\frac{\rho V_{\text{m}}}{2}\frac{{ɛ}_{0}}{kT}\;\left(1+\frac{53}{5}\frac{{ɛ}_{2}}{{ɛ}_{0}}\right)\right]$$

(31) $$a_{1}=-4C_{2}^{*}G(\eta )-\frac{\rho V_{\text{m}}}{2}\frac{{ɛ}_{0}}{kT}\;\left[4C_{2}^{*}\;\left(1+\frac{{ɛ}_{2}}{{ɛ}_{0}}\right)+6\frac{{ɛ}_{2}}{{ɛ}_{0}}\;\left(\frac{4\pi }{3}\frac{D^{3}}{V_{\text{m}}}-C_{0}^{*}\right)\right]$$

(32) $$a_{2}=-\frac{C_{1}^{*}\sqrt{\pi }}{2}\;\left[G(\eta )+\frac{\rho V_{\text{m}}}{2}\frac{{ɛ}_{0}}{kT}\;\left(1+\frac{{ɛ}_{2}}{{ɛ}_{0}}\right)\right]$$

The equilibrium value of *α* is determined by solving the cubic equation, the roots of which can be cast in a trigonometric form:

(33) $$\alpha_{j}=\frac{1}{9}\;{\left\{a_{2}-2\sqrt{a_{2}^{2}-3a_{1}}\text{cos }\left(\frac{2j\pi }{3}+\frac{1}{3}\text{arccos}\frac{-27\;[a_{0}-\frac{1}{3}a_{1}a_{2}+\frac{2}{27}a_{2}^{3}]}{2\;{\left[a_{2}^{2}-3a_{1}\right]}^{3/2}}\right)\right\}}^{2}$$

where *j* = 0, 1,2 represent the three solutions to Equation (29). The value of *α_j_*, describing the equilibrium orientational distribution of the nematic phase, corresponds to the largest root, (*j* = 0): *α*_eq_ = *α*_0_.

The expressions for the chemical potential, *μ*^nem^, and the compressibility factor (equation of state), *Z*^nem^ = *PV*/ (*NkT*), of the nematic phase can be derived from standard thermodynamic relationships, *μ* = (*∂A/∂N*)*_V_*_,_*_T_* and *P* = −( *∂A/∂V* )*_N_*_,_*_T_* :

(34) $$\begin{array}{l} \frac{\mu^{\text{nem}}}{kT}=\frac{\mu_{\text{iso}}^{\text{id}}}{kT}+\text{ln }\rho +\text{ln }\alpha_{\text{eq}}-1+\frac{1}{8}\frac{\mu_{\text{hs}}^{\text{res}}}{kT}\;\left[C_{0}^{*}+C_{1}^{*}\sqrt{\pi }\;\left(\frac{1}{\alpha_{\text{eq}}^{1/2}}-\frac{15}{16\alpha_{\text{eq}}^{3/2}}\right)+C_{2}^{*}\frac{4}{\alpha_{\text{eq}}}\right]\\ -\rho V_{\text{m}}\frac{{ɛ}_{0}}{kT}\{\left(\frac{4\pi }{3}\frac{D^{3}}{V_{\text{m}}}-C_{0}^{*}\right)\;\left(1+\frac{{ɛ}_{2}}{{ɛ}_{0}}\right)-C_{1}^{*}\sqrt{\pi }\;\left(1+\frac{{ɛ}_{2}}{{ɛ}_{0}}\right)\;\alpha_{\text{eq}}^{-1/2}\\ -\left[4C_{2}^{*}\;\left(1+\frac{{ɛ}_{2}}{{ɛ}_{0}}\right)+6\frac{{ɛ}_{2}}{{ɛ}_{0}}\;\left(\frac{4\pi }{3}\frac{D^{3}}{V_{\text{m}}}-C_{0}^{*}\right)\right]\;\alpha_{\text{eq}}^{-1}+\frac{15}{16}C_{1}^{*}\sqrt{\pi }\;\left(1+\frac{53}{5}\frac{{ɛ}_{2}}{{ɛ}_{0}}\right)\;\alpha_{\text{eq}}^{-3/2}\} \end{array}$$

and

(35) $$\begin{array}{l} Z^{\text{nem}}=1+\frac{Z_{\text{hs}}^{\text{res}}}{8}\;\left[C_{0}^{*}+C_{1}^{*}\sqrt{\pi }\;\left(\frac{1}{\alpha_{\text{eq}}^{1/2}}-\frac{15}{16\alpha_{\text{eq}}^{3/2}}\right)+C_{2}^{*}\frac{4}{\alpha_{\text{eq}}}\right]\\ -\frac{\rho V_{\text{m}}}{2}\frac{{ɛ}_{0}}{kT}\{\left(\frac{4\pi }{3}\frac{D^{3}}{V_{\text{m}}}-C_{0}^{*}\right)\;\left(1+\frac{{ɛ}_{2}}{{ɛ}_{0}}\right)-C_{1}^{*}\sqrt{\pi }\left(1+\frac{{ɛ}_{2}}{{ɛ}_{0}}\right)\;\alpha_{\text{eq}}^{-1/2}\\ -\left[4C_{2}^{*}\;\left(1+\frac{{ɛ}_{2}}{{ɛ}_{0}}\right)+6\frac{{ɛ}_{2}}{{ɛ}_{0}}\;\left(\frac{4\pi }{3}\frac{D^{3}}{V_{\text{m}}}-C_{0}^{*}\right)\right]\;\alpha_{\text{eq}}^{-1}+\frac{15}{16}C_{1}^{*}\sqrt{\pi }\;\left(1+\frac{53}{5}\frac{{ɛ}_{2}}{{ɛ}_{0}}\right)\;\alpha_{\text{eq}}^{-3/2}\} \end{array}$$

where $\mu_{\text{hs}}^{\text{res}}/kT=(3\eta^{3}-9\eta^{2}+8\eta )/{\left(1-\eta \right)}^{3}$ and $Z_{\text{hs}}^{\text{res}}=(4\eta -2\eta^{2})/{\left(1-\eta \right)}^{3}$ are the residual chemical potential and compressibility factor.

An important quantity that is used to characterize the degree of orientational order of the nematic phase is the nematic order parameter, *S*_2_, which is commonly defined as the orientational average of the second Legendre polynomial, *P*_2_(cos(*θ*)):

(36) $$S_{2}=\int P_{2}(\text{cos}(\theta ))f(\theta )\text{d}\vec{\omega }$$

Because the OTF is used to represent the orientational distribution in nematic phase, *f*(*θ*) = *f*_OTF_(*ω⃗*), *S*_2_ can be expressed as a function of the orientational parameter *α* [91]:

(37) $$S_{2}=1-\frac{3\;\text{coth }\alpha_{\text{eq}}}{\alpha_{\text{eq}}}+\frac{3}{\alpha_{\text{eq}}^{2}}$$

In the case of the isotropic phase, where no orientational order is exhibited, the orientational averages of Equation (14) are evaluated using the isotropic value, *f*(*ω⃗*) = 1/4*π*. The Helmholtz free energy expression for the isotropic phase is then given by:

(38) $$\begin{array}{l} \frac{A^{\text{iso}}}{NkT}=\frac{A_{\text{iso}}^{\text{id}}}{NkT}+G(\eta ){\langle \frac{V_{\text{exc}}(\gamma )}{V_{\text{m}}}\rangle }_{{\vec{\omega }}_{1},{\vec{\omega }}_{2}}^{\text{iso}}-\frac{\rho V_{\text{m}}}{2}\frac{{ɛ}_{0}}{kT}\;\left[\frac{4\pi }{3}\frac{D^{3}}{V_{\text{m}}}-{\langle \frac{V_{\text{exc}}(\gamma )}{V_{\text{m}}}\rangle }_{{\vec{\omega }}_{1},{\vec{\omega }}_{2}}^{\text{iso}}\right]\\ +\frac{\rho V_{\text{m}}}{2}\frac{{ɛ}_{2}}{kT}{\langle \frac{V_{\text{exc}}(\gamma )}{V_{\text{m}}}P_{2}(\text{sin }\gamma )\rangle }_{{\vec{\omega }}_{1},{\vec{\omega }}_{2}}^{\text{iso}} \end{array}$$

The orientational averages in the isotropic phase can be evaluated as:

(39) $${\langle \frac{V_{\text{exc}}(\gamma )}{V_{\text{m}}}\rangle }_{{\vec{\omega }}_{1},{\vec{\omega }}_{2}}^{\text{iso}}\approx C_{0}^{*}+\frac{\pi }{4}C_{1}^{*}+\frac{2}{3}C_{2}^{*}+\frac{8}{15}C_{4}^{*}$$

(40) $${\langle \frac{V_{\text{exc}}(\gamma )}{V_{\text{m}}}P_{2}(\text{sin }\gamma )\rangle }_{{\vec{\omega }}_{1},{\vec{\omega }}_{2}}^{\text{iso}}\approx -\frac{\pi }{32}C_{1}^{*}-\frac{2}{15}C_{2}^{*}-\frac{16}{105}C_{4}^{*}$$

From the expressions for free energy of the isotropic phase (cf., Equation (38)), we obtain the corresponding chemical potential and compressibility factor:

(41) $$\begin{array}{l} \frac{\mu^{\text{iso}}}{kT}=\frac{\mu_{\text{iso}}^{\text{id}}}{kT}+\text{ln }\rho +\frac{1}{8}\frac{\mu_{\text{hs}}^{\text{res}}}{kT}\;\left(C_{0}^{*}+\frac{\pi }{4}C_{1}^{*}+\frac{2}{3}C_{2}^{*}+\frac{8}{15}C_{4}^{*}\right)\\ -\rho V_{\text{m}}\frac{{ɛ}_{0}}{kT}\;\left(\frac{4\pi }{3}\frac{D^{3}}{V_{\text{m}}}-C_{0}^{*}-\frac{\pi }{4}C_{1}^{*}-\frac{2}{3}C_{2}^{*}-\frac{8}{15}C_{4}^{*}\right)\\ +\rho V_{\text{m}}\frac{{ɛ}_{2}}{kT}\;\left(-\frac{\pi }{32}C_{1}^{*}-\frac{2}{15}C_{2}^{*}-\frac{16}{105}C_{4}^{*}\right) \end{array}$$

and

(42) $$\begin{array}{l} Z^{\text{iso}}=1+\frac{Z_{\text{hs}}^{\text{res}}}{8}\;\left(C_{0}^{*}+\frac{\pi }{4}C_{1}^{*}+\frac{2}{3}C_{2}^{*}+\frac{8}{15}C_{4}^{*}\right)\\ -\frac{\rho V_{\text{m}}}{2}\frac{{ɛ}_{0}}{kT}\;\left(\frac{4\pi }{3}\frac{D^{3}}{V_{\text{m}}}-C_{0}^{*}-\frac{\pi }{4}C_{1}^{*}-\frac{2}{3}C_{2}^{*}-\frac{8}{15}C_{4}^{*}\right)\\ +\frac{\rho V_{\text{m}}}{2}\frac{{ɛ}_{2}}{kT}\;\left(-\frac{\pi }{32}C_{1}^{*}-\frac{2}{15}C_{2}^{*}-\frac{16}{105}C_{4}^{*}\right) \end{array}$$

Before proceeding, we should note that the PL approach for the isotropic-nematic phase of hard disc-like particles has been compared with a generic equation of state, which accounts for both negative and positive contributions of the higher-body virial coefficients [46]; only positive virial coefficients are possible with the PL approximation. Though an improved quantitative description of the isotropic-nematic coexistence densities when compared with the exact simulation data is obtained by incorporating the negative virial coefficients (particularly for the very thin discs), both approaches provide the same overall qualitative fluid-phase behaviour. A comparison of the differences in the two approaches for our systems of attractive hard discs is made in the following section.

## 3. Results and Discussion

The fluid-phase diagrams of attractive cylindrical discs of various aspect ratios with square-well attractive interaction are calculated by equating the pressure and chemical potential of the coexisting phases at a given temperature. For the ACD model, the range of the attractive potential is characterized by the parameter *λ*, which is chosen to be *λ* ≥ 1 in order to ensure that the resulting integrals for the contributions from the repulsive and attractive interactions remain separable. Dimensionless units are adopted throughout: pressure, P* = *PV*_m_/*ε*_0_, and packing fraction, *η* = *ρV*_m_. Two dimensionless scales for the temperature have been used in our study: the dimensionless temperature, T* = *kT/ε*_0_, in terms of the isotropic square-well depth, *ε*_0_; and the reduced van der Waals-like temperature, $T_{\text{vdw}}^{*}=kT/\lambda^{3}$. Though the dimensionless form, T*, is commonly used to denote the temperature in simulations of such systems, the reduced van der Waals temperature, $T_{\text{vdw}}^{*}$, is useful to allow for direct comparisons in terms of the strength of the attractive interactions (corresponding states representation).

### 3.1. Attractive Cylindrical Discs with Isotropic SW Potentials

**Figure 2:**
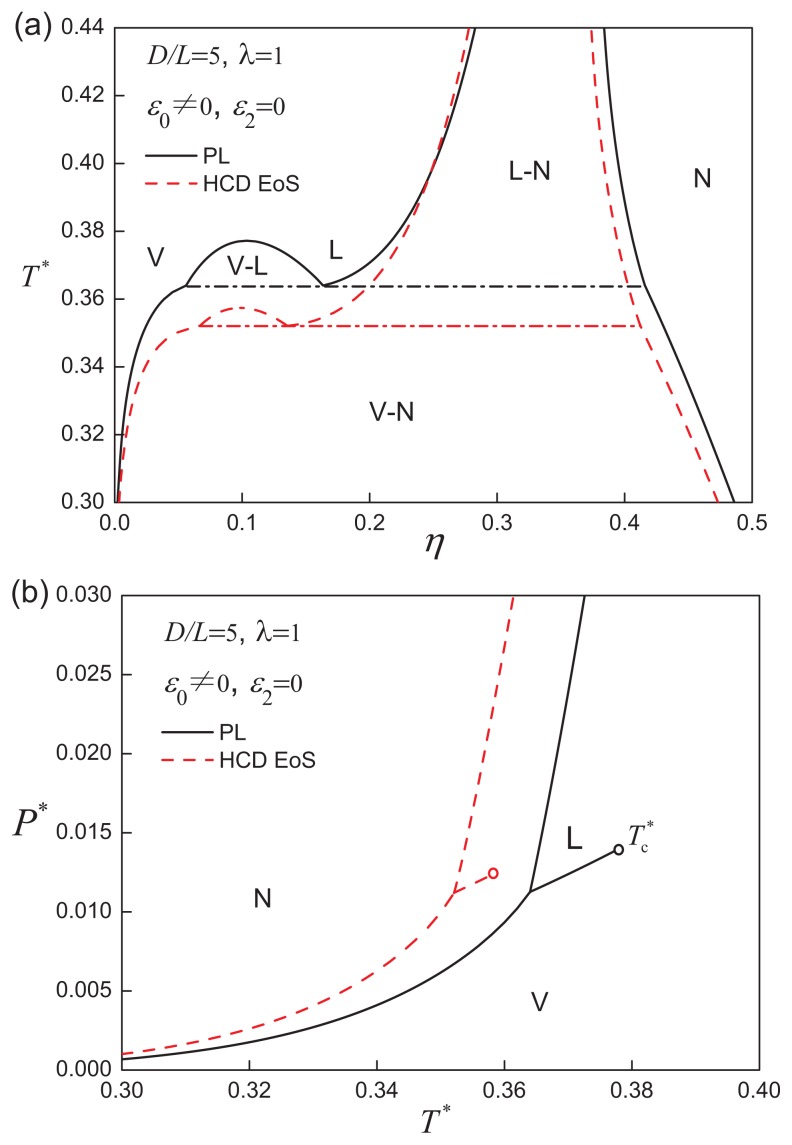
(**a**) The temperature-density and (**b**) pressure-temperature representations of the fluid-phase equilibria for attractive cylindrical discs (ACDs) with an aspect ratio of *D/L* = 5 and an isotropic attractive interaction of range *λ* = 1. The generic disc-like molecules are modelled as hard cylinders of length *L* and diameter *D*, enveloped by an attractive square well of depth −(*ε*_0_ + *ε*_2_*P*_2_(cos *γ*)) and range *λD*; in the case of isotropic attractions, *ε*_0_ ≠ 0 and *ε*_2_ = 0. The dimensionless properties are defined in terms of *D* and *ε*_0_, as T* = *kT/ε*_0_ for the temperature, P* = *PD*^3^*/ε*_0_ for the pressure and *η* = *ρV_m_* for the packing fraction, where *V_m_* is the volume of the cylindrical core. The stable phases are indicated as vapour (V), isotropic liquid (L) and nematic (N), and $T_{\text{c}}^{*}$ denotes the vapour-liquid equilibrium (VLE) critical point. The dashed curves represent the results obtained using a generic reference EOS for hard-cylindrical discs (HCDs) [46]. The dot-dashed line in (**a**) represents the V-L-N three-phase coexistence line.

First, we focus on discs with a spherically symmetric SW potential, so that the orientation-dependent attractive contribution is inactive, *i.e*., *ε*_2_ = 0. Although the attractive interaction is isotropic, the orientation-dependent excluded volume gives rise to an overall interaction between the discs, which is anisotropic. The fluid-phase behaviour of ACDs of aspect ratio *D/L* = 5 and attractive range *λ* = 1 is presented in Figure 2. It is clear from the phase diagram that the system exhibits three fluid phases: vapour (V), liquid (L), and nematic (N). The vapour-liquid equilibrium (VLE), seen at relatively low densities, which terminates at the VLE critical point, ( $T_{\text{c}}^{*}=0.37720$, *η*_c_ = 0.10362 and $P_{\text{c}}^{*}=0.01383$), is determined by the free energy of the isotropic phase of the ACD particles. Oblate hard cores with square-well attractive interactions have been studied by Meneses-Juárez *et al*. [92], but in this case the range of the attractive interaction is smaller than the diameter of the molecule, so a direct comparison is not possible. At moderate to high densities, an isotropic liquid-nematic transition is observable. In the low temperature region, the isotropic-nematic coexistence becomes a broad region of vapour-nematic equilibrium (VNE), which is bounded by a vapour-liquid-nematic (V-L-N) triple line. Above the triple temperature, the nematic phase coexists with an isotropic liquid phase. It is worthwhile noting that the width of liquid-nematic equilibrium (LNE) coexistence curve broadens markedly when *T* approaches the triple point.

**Figure 3:**
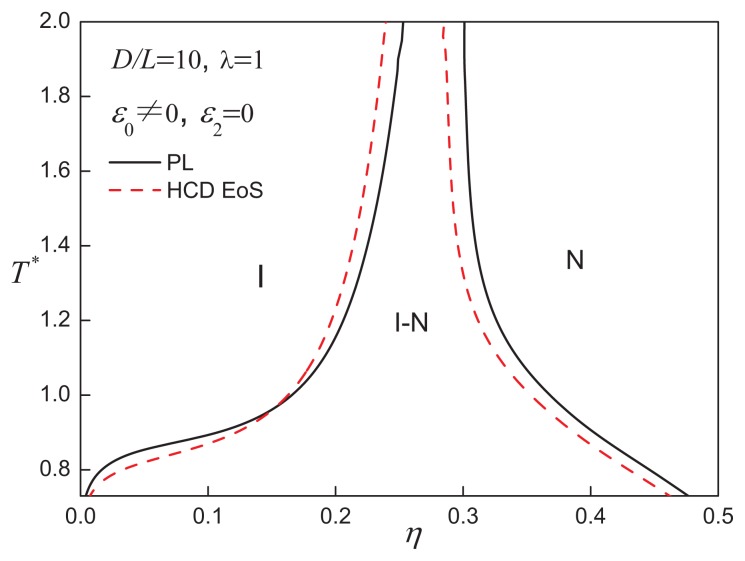
Temperature-density representation of the fluid-phase equilibria for attractive cylindrical discs (ACDs) with an aspect ratio of *D/L* = 10 and an isotropic attractive interaction of range *λ* = 1. The stable phases are indicated as isotropic (I) and nematic (N). See the caption of Figure 2 for further details.

The model developed in our current work allows one to assess the effect of the anisotropy of the underlying hard disc on the phase behaviour of the ACD system. The phase diagram of hard discs of aspect ratio *D/L* = 10 with attractive interactions of range *λ* = 1 is shown in Figure 3. The system shows a single region of coexistence between isotropic (vapour or liquid) and nematic phases within the temperature range considered. For the attractive disc systems with an aspect ratio of *D/L >* 5, the VLE becomes metastable with respect to isotropic-nematic (I-N) coexistence. The region of I-N coexistence is broad at low temperatures, with the difference between the coexisting densities of the isotropic and nematic phases becoming narrow at higher temperatures. The coexisting densities of the two phases is also seen to gradually shift to higher packing fractions when the temperature is increased. The contribution of the attractive interactions stabilizes the orientational order in the low-density and low-temperature region. The I-N transition of the attractive disc model approaches the behaviour of the corresponding purely repulsive discs in the high-temperature region.

**Figure 4:**
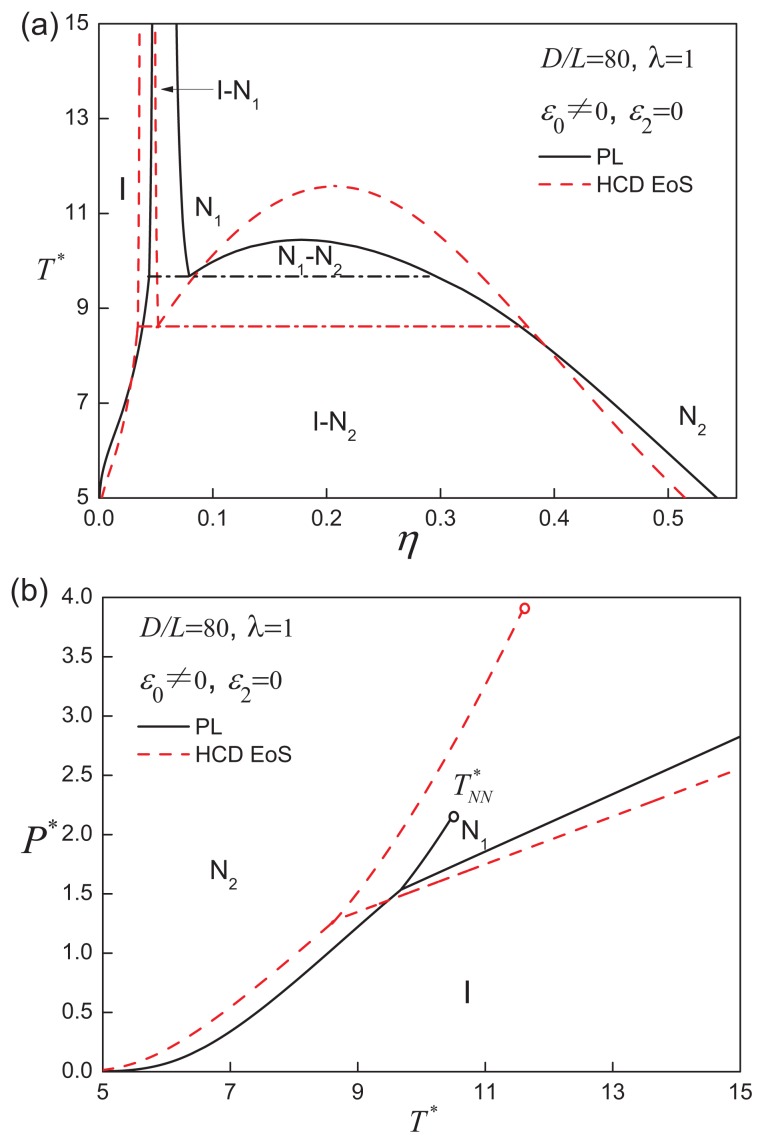
(**a**) The temperature-density and (**b**) pressure-temperature representations of the fluid-phase equilibria for attractive cylindrical discs (ACDs) with an aspect ratio of *D/L* = 80 and an isotropic attractive interaction of range *λ* = 1. The stable phases are indicated as isotropic liquid (I), low-density nematic (N_1_) and high-density nematic (N_2_), and $T_{\text{NN}}^{*}$ is the nematic-nematic critical point. The dot-dashed line in (**a**) represents the I-N_1_-N_2_ three-phase coexistence line. See the caption of Figure 2 for further details.

**Figure 5:**
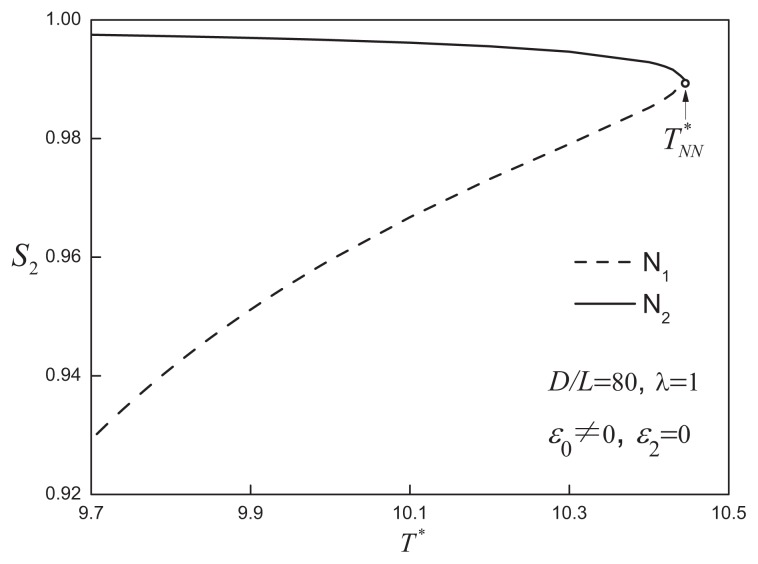
The temperature dependence of the nematic order parameter, *S*_2_, of the coexisting nematic phases for attractive cylindrical discs (ACDs) of aspect ratio *D/L* = 80 with an isotropic attractive interaction of range *λ* = 1 (cf., Figure 4) The nematic-nematic critical point, $T_{\text{NN}}^{*}$, is also indicated. See the caption of Figure 2 for further details.

When the aspect ratio is increased to *D/L* = 80, a different type of phase behaviour is exhibited by the system. As shown in Figure 4, the isotropic-nematic coexistence is located at low densities (*η* ~ 0.1), while a region of nematic-nematic equilibrium (NNE) appears at moderate to high densities. The N_1_-N_2_ coexistence is bounded by the I-N_1_-N_2_ triple line and nematic-nematic critical point ( $T_{\text{NN}}^{*}$). An examination of the temperature dependence of the nematic order parameter is shown in Figure 5: the degree of order of the high-density nematic state (N_2_) decreases slightly, while that of the lowdensity nematic state (N_1_), though still highly ordered, behaves as a monotonically increasing function of temperature up to $T_{\text{NN}}^{*}$. At $T_{\text{NN}}^{*}$, by definition, the differences in density and orientational order between two nematic phases become indistinguishable.

As the aspect ratio of the discs is made larger (corresponding to thinner particles), the isotropic-nematic moves to lower densities. For particles that are moderately thin (*D/L* = 10 to 50), the vapour-liquid transition between the two isotropic fluid phases becomes metastable with respect to a transition between an isotropic liquid and a nematic phase. For larger aspect ratios (e.g., *D/L >* 80), the isotropic-nematic transition moves to very low densities (packing fractions below 10%). As a consequence, the usual van derWaals “vapour-liquid” phase transition is now exhibited in the anisotropic region of the phase diagram, corresponding to nematic-nematic coexistence with its associated critical point. A similar progression from V-L-N, through I-N, to I-N_1_-N_2_ phase behaviour with increasing aspect ratio has been observed for attractive rod-like LC molecules [77,93–98]. The coexistence between two nematic phases has been found experimentally in studies of hexa-alkylbenzene derivatives of discotic mesogens [99] and in solutions of the calamitic polypeptides [95], both of which are characterized by extreme oblate and prolate aspect ratios. It is also interesting to note that an analogous transition in the vapour-liquid-solid phase equilibria is exhibited by attractive spherical particles as the range of interaction is decreased: the VLE is found to become metastable relative to the isotropic fluid-solid transition, and on further decreasing the attractive range, an iso-structural coexistence between two solid phases is observed [100,101].

Also included in Figures 2, 3 and 4 are calculations using an improved representation of the reference EOS [46] of the isotropic and nematic states of hard-cylindrical discs HCDs. It is clear that the qualitative features of the fluid-phase behaviour are similar to those obtained with the PL approach for the hard-core reference system, so we retain the standard PL treatment for simplicity, and the HCD reference EOS is not employed in the subsequent analysis.

**Figure 6:**
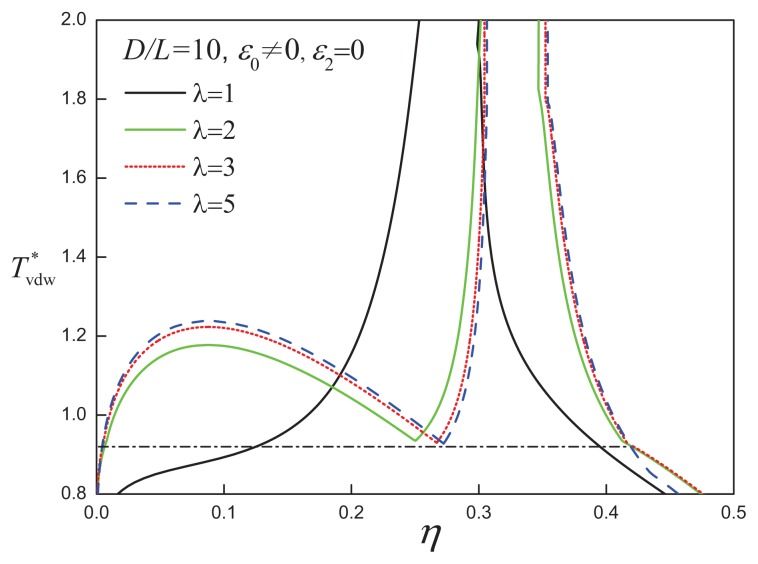
Temperature-density representation of the fluid-phase equilibria of attractive cylindrical discs (ACDs) with an aspect ratio *D/L* = 10 and isotropic attractive interaction of varying range, *λ*. The dot-dashed line corresponds to the V-L-N three-phase coexistence line for the systems with *λ* = 3 and 5. The reduced temperature is defined in a van der Waals-corresponding states form as $T_{\text{vdw}}^{*}=T^{*}/\lambda^{3}$. See the caption of Figure 2 for further details.

By varying the parameter *λ*, different ranges of the attractive interaction may be explored. The temperature-density projections of the fluid-phase equilibria are shown in Figure 6 for discs with an aspect ratio of *D/L* = 10 and attractive ranges of *λ* = 1, 2, 3 and 5 in a representation where the temperature is reduced by the attractive range, $T_{\text{vdw}}^{*}=T^{*}/\lambda^{3}$. As the attractive range is increased, the phase diagrams for the *D/L* = 10 discs with *λ* = 3 and 5 exhibit vapour, liquid, and nematic phases with corresponding triple points, the same features as seen with the less anisotropic discs (*D/L* = 5, *λ* = 1). The fluid-phase coexistence of ACDs with *λ* = 3 and 5 converge onto a universal van der Waalsian curve. However, it is apparent that the phase coexistence curve for *λ* = 1 does not follow this type of corresponding state behaviour. Presumably, the VLE coexistence is not observed for attractive discs with the same aspect ratio (*D/L* = 10), but a smaller attractive range (*λ* = 1), because the average excluded volume of a pair of discs is of comparable size to the enveloping attractive sphere of the isotropic SW interaction.

**Figure 7:**
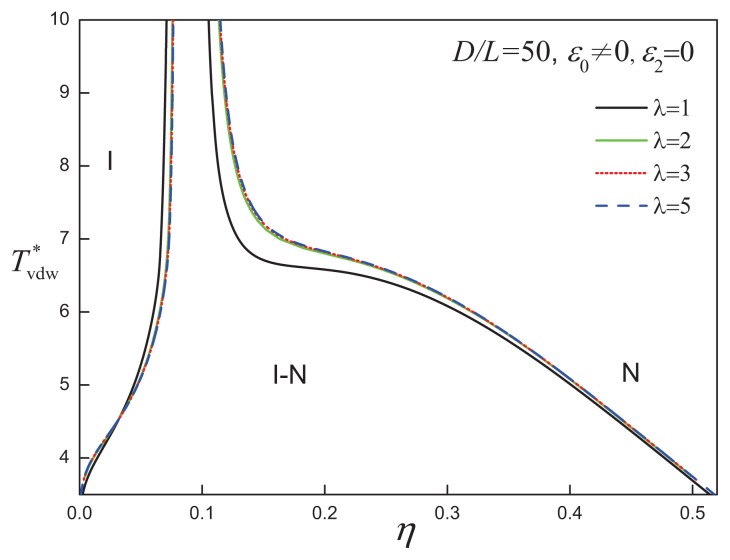
Temperature-density representation of the fluid-phase equilibria of attractive cylindrical discs (ACDs) with an aspect ratio *D/L* = 50 and isotropic attractive interaction of varying range, *λ*. The reduced temperature is defined in a van der Waals-corresponding states form as $T_{\text{vdw}}^{*}=T^{*}/\lambda^{3}$. See the caption of Figure 2 for further details.

**Figure 8:**
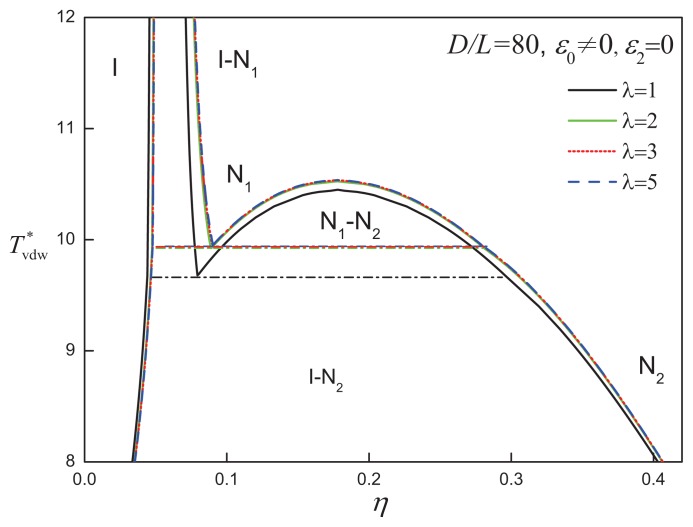
Temperature-density representation of the fluid-phase equilibria of attractive cylindrical discs (ACDs) with an aspect ratio *D/L* = 80 and isotropic attractive interaction of varying range, *λ*. The reduced temperature is defined in a van der Waals-corresponding states form as $T_{\text{vdw}}^{*}=T^{*}/\lambda^{3}$. The dot-dashed lines correspond to the I-N_1_-N_2_ three-phase coexistence lines in each case. See the caption of Figure 2 for further details.

**Figure 9:**
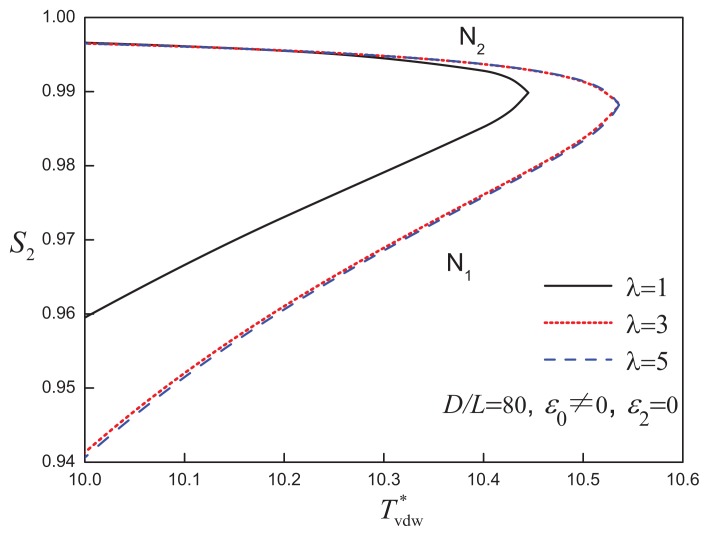
The temperature dependence of the nematic order parameter, *S*_2_, of the coexisting nematic phases for attractive cylindrical discs (ACDs) of aspect ratio *D/L* = 80 with an isotropic attractive interaction of varying range, *λ* (cf., Figure 8) The nematic-nematic critical point, $T_{\text{NN}}^{*}$, is also indicated. See the caption of Figure 2 for further details.

The fluid-phase behaviour of discs characterized by an aspect ratio *D/L* = 50 is shown in Figure 7 for various values of the attractive range. It is noted that the large attractive range widens the isotropicnematic coexistence and shifts the I-N transition to higher densities. Increasing the attractive range is not seen to promote a change in phase behaviour, supporting the view that the hard-body interaction (free-volume entropy) is the dominant feature of highly anisometric particles. The coexistence between two nematic phases is maintained in attractive disc systems of even larger aspect ratio, *D/L* = 80. In Figure 8, systems of *D/L* = 80 attractive discs of varying attractive range are seen to exhibit the same type of phase diagram, and all appear to obey a corresponding state principle in terms of the reduced temperature. Although a larger range of the isotropic attraction does not qualitatively affect the phase behaviour of discs with *D/L* = 80, the degree of orientational order of the coexisting low-density nematic phase (N_1_) becomes lower as *λ* increases, as can be seen in Figure 9.

These examples indicate that the long-ranged isotropic SW attraction slightly destabilizes the orientationally-ordered phases. In the case of the thicker discs (*D/L* = 10), the long-range attractions (*λ* = 3 and 5) promote the coexistence between gas and isotropic liquid phases with no orientational order (*S*_2_ = 0), while attractive discs with short-ranged attractive interactions (*λ* = 1) exhibit an isotropic-nematic transition. For the systems with large aspect ratios (*D/L* = 50 and 80), where the phase behaviour is dominated by the excluded volume interactions, the long-range isotropic attractions do not change the type of phase behaviour, but weaken the degree of the orientational order of the coexisting nematic phases.

### 3.2. Attractive Cylindrical Discs with Anisotropic SW Potentials

**Figure 10:**
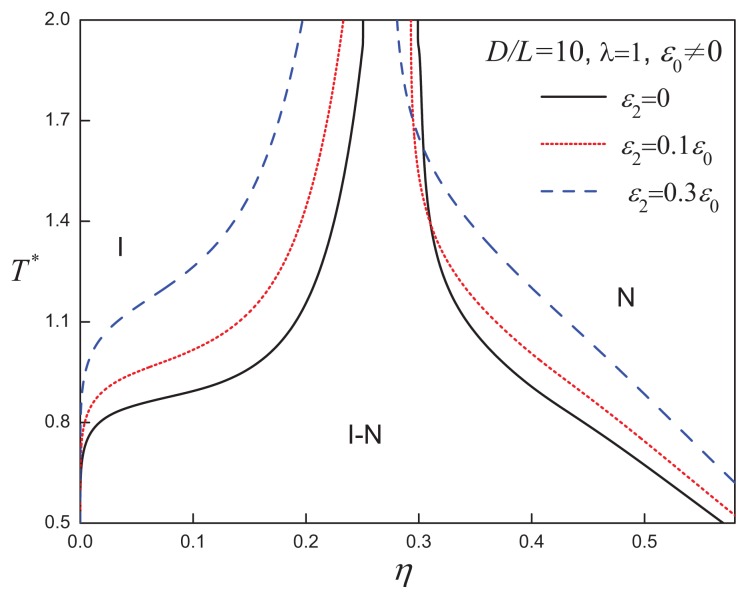
Temperature-density representation of the fluid-phase equilibria of attractive cylindrical discs (ACDs) with an aspect ratio of *D/L* = 10, and a positive anisotropic attractive interaction range *λ* = 1 and varying strength *ε*_2_*>* 0. See the caption of Figure 2 for further details.

**Figure 11:**
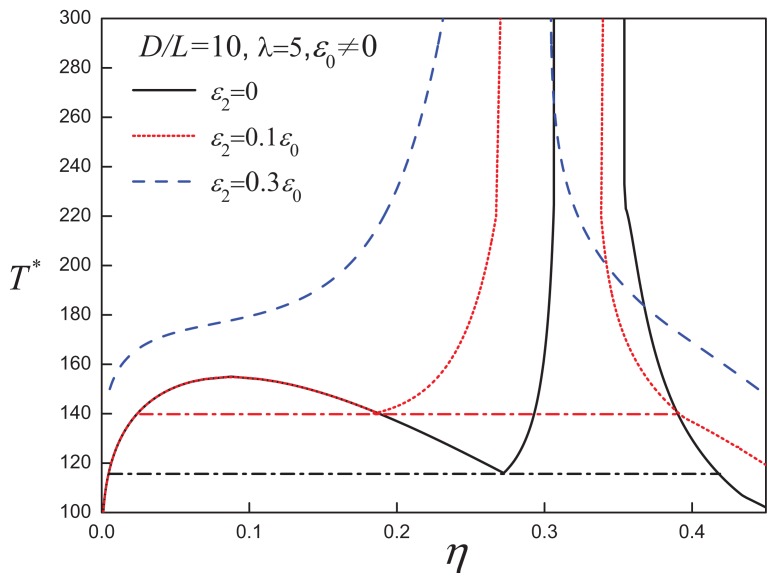
Temperature-density representation of the fluid-phase equilibria of attractive cylindrical discs (ACDs) with an aspect ratio of *D/L* = 10 and an positive anisotropic attractive interaction range, *λ* = 5, and varying strength, *ε*_2_*>* 0. The dot-dashed lines corresponds to the V-L-N three-phase coexistence line in each case. See the caption of Figure 2 for further details.

In real systems, the dispersion forces are associated with the functional groups distributed at various points in the molecule. The assignment of a central isotropic attraction is but a simplistic first-order approximation. Recognizing the morphological anisotropy of discotic LCs, one can postulate the existence of additional orientation-dependent (anisotropic) attractions. The effect of including anisotropic attractions on the fluid-phase diagram of discs with *D/L* = 10 and *λ* = 1 is shown in Figure 10: increasing the anisotropy of the attractions does not alter the phase behaviour qualitatively, but drives the I-N transition to lower densities. The VLE in the low-density region is very sensitive to the incorporation of anisotropic attractive interactions. As shown in Figure 11, the VLE is destabilized on increasing the strength of the positive anisotropic attractions; for a sufficient large value of the anisotropy, *ε*_2_ = 0.3*ε*_0_, the VLE becomes metastable with respect to the isotropic-nematic coexistence. A similar phenomena was also reported for the systems of attractive spherical and rod-like particles with anisotropic attractions [77].

**Figure 12:**
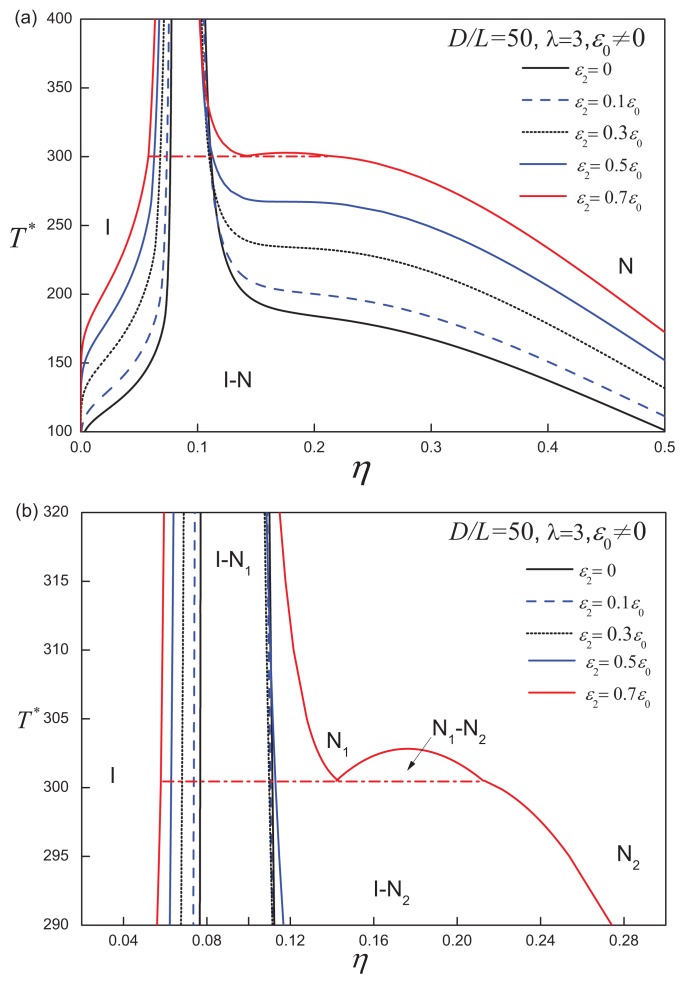
(**a**) Temperature-density representation of the fluid-phase diagram for ACD with an aspect ratio of *D/L* = 50, attractive range *λ* = 3 and varying anisotropic interaction strength *ε*_2_. The dot-dashed line represents th I-N_1_-N_2_ three-phase coexistence line; (**b**) The nematic-nematic coexistence region for the case of *ε*_2_ = 0.7*ε*_0_. See the caption of Figure 2 for further details.

It is clear that a positive anisotropic attractive interaction stabilizes the nematic phase and enhances the propensity of the system to form orientationally-ordered states. For a system of discs with a large aspect ratio, e.g., *D/L* = 50 (cf., Figure 12), the isotropic-nematic region becomes much broader at lower temperatures as *ε*_2_ is increased, while the system converges into the hard-disc limit in the high-temperature limit. For highly anisometric systems, the anisotropic shape and orientation-dependent attractions both contribute to the stabilization of the nematic phases. Hence, for an anisotropic strength of *ε*_2_ = 0.7*ε*_0_, which is comparable to the isotropic attraction, the *D/L* = 50 discs with anisotropic attractions exhibit a nematic-nematic coexistence, which is equivalent to that seen for *D/L* = 80 discs with isotropic attractions.

**Figure 13:**
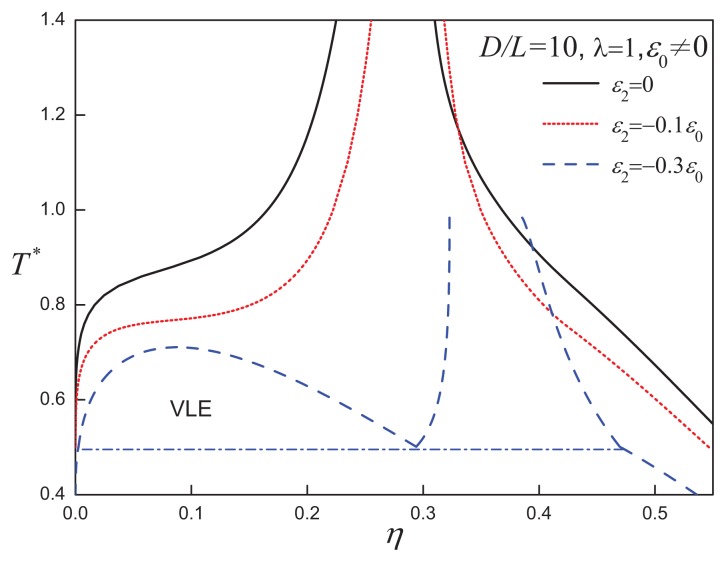
Temperature-density representation of the fluid-phase equilibria of attractive cylindrical discs (ACDs) with an aspect ratio of *D/L* = 10 and a negative anisotropic attractive interaction of range *λ* = 1 and varying strength, *ε*_2_*<* 0. The dot-dashed line corresponds to the V-L-N three-phase coexistence line. See the caption of Figure 2 for further details.

**Figure 14:**
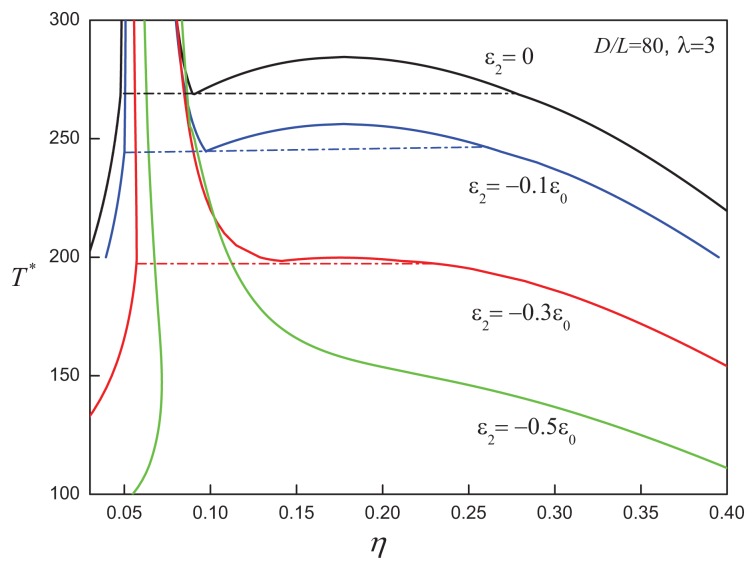
Temperature-density representation of the fluid-phase equilibria of attractive cylindrical discs (ACDs) with an aspect ratio of *D/L* = 80 and an negative anisotropic attractive interaction of range *λ* = 3 and varying strength, *ε*_2_*<* 0. The dot-dashed lines correspond to the I-N_1_-N_2_ three-phase coexistence lines in each case. See the caption of Figure 2 for further details.

In addition to attractive interactions with a positive anisotropy, for some molecules one would expect interactions with a negative anisotropy favouring a perpendicular configuration: e.g., the quadrupolar interactions between aromatic moieties give rise to both parallel (side-by-side) and perpendicular (Tshaped) relative orientations of the cores. These kinds of interactions can be represented in our ACD models using negative values for the parameter *ε*_2_. In Figures 13 and 14, we present the phase behaviour of *D/L* = 10, *λ* = 1 and *D/L* = 80, *λ* = 3 disc systems, respectively. In the case of the *D/L* = 10, *λ* = 1 system, increasing the negative anisotropic attractions leads to a destabilization of the isotropic-nematic region and promotes the appearance of VLE when *ε*_2_ = −0.3*ε*_0_. For discs with a larger aspect ratio of *D/L* = 80, the orientation-dependent attractive interaction is seen to destabilize the nematic-nematic equilibrium and to drive the isotropic phase boundary to higher densities. For sufficiently large values of the negative anisotropic interaction, *ε*_2_ = −0.5*ε*_0_, the system no longer exhibits the region of nematic-nematic coexistence seen for the equivalent system with less negative anisotropic interaction, *ε*_2_*>* −0.3 (cf., Figure 14).

## 4. Conclusions

In this work, we present a closed-form equation of state for the description of the thermodynamic properties and orientational ordering of attractive hard-core cylindrical-disc fluids, which serves as a basic model for thermotropic discotic liquid crystals. With the aid of the Onsager trial function to represent the single-particle orientational distribution function in the nematic phase, the free energy is expressed in algebraic form and the functional variation of free energy reduced to a simple derivative with respect to the Onsager orientational parameter *α*. A cubic solution of *α*_eq_ is then obtained when higher-order terms in the expansion are neglected and ordered states (*α*~10) are considered. Using this approach, we provide a qualitative description of the fluid-phase diagrams of ACD particles. The separate effects of the shape anisotropy of the hard discs and the orientation-dependent attractive interactions are examined in detail. The hard core of the particle is primarily responsible for determining the type of phase behaviour. The contribution of attractive interactions, though secondary, cannot be neglected in thermotropic LC systems. For the ACD model, the attractive range, *λ*, of the enveloping square well determines the formation of the isotropic liquid state. The anisotropic term in the ASW potential, which is controlled by the coefficient *ε*_2_ (or its ratio *ε*_2_*/ε*_0_ relative to the isotropic term), is key to the stabilization/destabilization of orientationally-ordered phases. The attractive disc model studied in the current work is a simple prototypical coarse-grained representation of real molecular interactions in discotic liquid crystals: neither will the repulsive interactions be purely repulsive in real systems nor will the attractive interactions be of the simple van der Waals square-well form. This having been said, many common discotic thermotropic particles comprise large fused aromatic cores, which will have lower energetic overlap volumes at the edges of the particles than in the central region, which is captured volumetrically at least with our square-well model. We are therefore confident that, qualitatively, at least, our model will describe the isotropic and nematic ordering behaviour of discotic thermotropic mesogens. A simple square-well hard-spherocylinder model of this generic form has been used to successfully represent the ordering behaviour of solutions of rod-like polypeptide (poly *γ*-benzyl L-glutamate) macromolecules with a quantitative description of the phase boundaries [98].

It is important to point out that in liquid state theory [49], any perturbation approach requires an accurate description of the reference system. The reference adopted here for the ACDs is the hard-cylindrical disc system described with a Parsons-Lee approach. Because of anomalous negative contributions from higher-order virial coefficients [42], the Parsons-Lee approach does not provide an accurate description of hard-disc fluids, when compared with the exact simulation data, particularly in the limit of infinitely thin discs. An improved equation of state for oblate disc-like particles has been developed [46], which incorporates these contributions and provides a better description for the isotropic and nematic phases of hard-disc systems. In the current work, we have not treated the columnar states that are expected in the high density region of the phase diagram.

The methodology developed here allows other interactions to be incorporated within the attractive hard-disc to provide a more realistic free energy functional. The molecular-based statistical associating fluid theory (SAFT) [102,103], and its recent extensions [104–108], have been shown to be a powerful methodology, allowing accurate descriptions of the phase behaviour of complex fluids and fluid mixtures. By building on the SAFT formalism, it is possible to explicitly include additional perturbation terms, such as those related to chain (or ring) geometries, hence paving the path towards a more sophisticated model for disc-like LC molecules. The use of the attractive disc model to describe the LC phase behaviour of real disc-like molecular fluids and the extension of the current methodology to other anisotropic phases (e.g., columnar ordering) will be the subject of future studies.
